# A case of aortitis and saccular aortic aneurysm caused by rare aetiological organisms, *Streptococcus constellatus* and parvovirus B19, and atherosclerosis

**DOI:** 10.1093/omcr/omae100

**Published:** 2024-09-02

**Authors:** Aly Amer, Neil Mangrolia, Ravi De Silva

**Affiliations:** Cardiology Department, East Suffolk and North Essex NHS Foundation Trust, Heath Road, Ipswich, Suffolk IP4 5PD, United Kingdom; Cardiology Department, East Suffolk and North Essex NHS Foundation Trust, Heath Road, Ipswich, Suffolk IP4 5PD, United Kingdom; Department of Cardiothoracic Surgery, Royal Papworth Hospital NHS Foundation Trust, Papworth Road Cambridge Biomedical Campus, Cambridge, Cambridgeshire CB2 0AY, United Kingdom

**Keywords:** aortitis, saccular aortic aneurysm, S. constellatus, parvovirus B19, atherosclerosis

## Abstract

Aortitis and mycotic aneurysm are vascular conditions characterized by inflammation of the aortic wall or the presence of an aneurysm resulting from microbial infection. This is a rare case of aortic aneurysm caused by atherosclerosis, with *Streptococcus constellatus* and Parvovirus B19 infection, in a 60-year-old male. The patient presented with rigors and pleuritic chest pain, and was found to have a saccular aneurysm of the ascending aorta and pericardial effusion. The patient underwent urgent replacement of the ascending aorta and completed 6 weeks of antibiotics with good recovery. This case emphasizes the importance of considering rare organisms in patients with aortitis and mycotic aneurysm, particularly in cases with blood cultures without microbial growth. Early diagnosis and treatment may be essential for the prevention of life-threatening complications.

## Introduction

Aortitis and mycotic aneurysm are vascular conditions characterized by inflammation of the aortic wall, or the presence of an aneurysm resulting from microbial infection [1]. Aortic aneurysms are classified based on their location: abdominal aortic aneurysms (AAA) and thoracic aortic aneurysms (TAA). AAA is more common, particularly in older adults, with a prevalence of about 4%–8% in men over 60 years old, whereas TAA has an incidence of about 10 per 100 000 person-years [2, 3]. Inflammatory aortic aneurysms, which account for a smaller subset, involve conditions such as Takayasu arteritis, giant cell arteritis, Behçet disease, and rheumatoid arthritis [4]. Infectious aortic aneurysms, though rare, are often caused by pathogens like *Staphylococcus aureus*, Salmonella, and less commonly, *S. constellatus* and Parvovirus B19 [5]. Diagnosis relies on laboratory and imaging findings, with blood cultures often considered the gold standard for identifying causative microorganisms. However, this report details a case caused by atherosclerosis and rare causative organisms, *S. constellatus* and Parvovirus B19, which were not detected by blood cultures. It underscores the importance of considering unusual microorganisms and highlights the value of employing alternative diagnostic modalities, such as serologic and molecular techniques.

## Case presentation

A 60-year-old male of Bangladeshi origin presented to our hospital with a 15-day history of rigors and chest pain radiating to the back. He was an ex-smoker with a history of diabetes and hypertension. He reported arthralgia and stiffness localized to the distal interphalangeal joints of both middle fingers.

On admission, his electrocardiogram was normal. Inflammatory markers were elevated, with a CRP of 265 mg/l and a white blood cell count (WBC) of 12.1 × 10^9^/l. D-dimer was markedly elevated at 5836 ng/ml. Troponin levels were normal.

**Figure 1:**
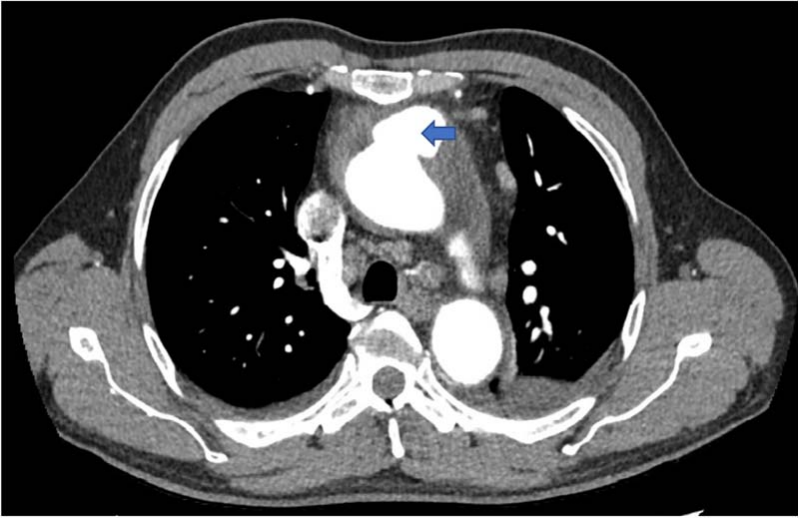
Axial CT image showing saccular aneurysm of the ascending aorta.

A computed tomography (CT) aortic angiogram was performed to exclude aortic dissection. A CT angiogram showed a saccular aneurysm of the ascending aorta and a pericardial effusion (Fig. 1). The aneurysm measured 2.8 cm in maximum diameter with a neck of 1.8 cm, and extended into the pericardium where there was an associated effusion with a maximal depth of 3 cm. The density of this effusion was 20 Hounsfield Units consistent with high protein content. Echocardiography revealed the presence of a moderate pericardial effusion with no diastolic cardiac chamber collapse, normal left ventricular function and unremarkable cardiac valves. Three sets of blood cultures showed no microbial growth. A syphilis screen was performed in view of the aneurysm and was negative.

In response to the patient’s clinical presentation and elevated inflammatory markers, empiric antibiotic therapy with co-amoxiclav and clarithromycin was commenced to address the suspected infectious etiology. A multidisciplinary discussion (MDT) was conducted with cardiology and cardiothoracic surgery, leading to transfer to the Royal Papworth Hospital for urgent surgery of the ascending aorta.

Preoperatively, he received meropenem and vancomycin. During the operation, samples were taken and sent to microbiology and histopathology. He underwent replacement of the ascending aorta for a pseudoaneurysm and there were contained ruptured dense intrapericardial adhesions and fibrinous deposit over the heart and great vessels. The patient had a large contained rupture sac, consisting only of aortic adventitia, and a small orifice into the false aneurysm sac.

The samples taken during surgery revealed that his aortic tissue culture PCR result was positive for *S. constellatus*. His blood serology showed IgG and IgM Parvovirus B-19. *S. constellatus* cultured from the patient was sensitive to penicillin (MIC: 0.047 μg/ml) and vancomycin but showed intermediate resistance to gentamicin (MIC: 8.0 μg/ml).

Histopathological examination confirmed the presence of a pseudoaneurysm within the adventitia, containing fibrin and abundant neutrophil polymorphs suggestive of an abscess. There was evidence of disrupted medial elastic fibers and focal dissection within the outer media. Necrosis and neutrophil infiltration were present at the breach site, with moderate atherosclerosis and background medial degeneration. The pericardium exhibited organizing fibrinous pericarditis.

Following surgery, he was transferred back to Ipswich hospital for 6 weeks of i.v. benzylpenicillin and made a good recovery. Postoperative echocardiography showed a small pericardial effusion measuring up to 1.4 cm around the inferolateral segment of the left ventricle and 1.1 cm around the right ventricular free wall. There was mild biventricular impairment.

## Discussion

The *Streptococcus anginosus* group (SAG) consists of three bacteria (*Streptococcus intermedius*, *S. constellatus*, and *S. anginosus*) that are known commensals of the upper respiratory, digestive, and reproductive tracts. While a rare occurrence, these bacteria have the capability of causing devastating pyogenic infections and ensuing abscess formations [6]. In this patient, the organism likely played a significant role in the development of aortitis and subsequent aneurysm, possibly facilitated by pre-existing atherosclerosis.Parvovirus B19, primarily known for causing erythema infectiosum in children, has also been implicated in various cardiovascular diseases, including myocarditis and endocarditis. Its presence in this case suggests a possible contributory role in the inflammatory process leading to aortic damage. While the exact mechanism by which Parvovirus B19 affects the cardiovascular system remains unclear, it is hypothesized that the virus can induce endothelial cell injury and inflammatory responses that may contribute to vascular pathology [7]. This case report highlights the role of *S. constellatus* and Parvovirus B19 as contributory causative agents for aortitis and mycotic aneurysm. Molecular techniques (PCR) and serologic tests played a pivotal role in identifying *S. constellatus* and Parvovirus B19 when blood cultures were uninformative [8]. Due to the critical nature of the patient’s condition, urgent replacement of the ascending aorta was necessary.

Histopathological examination of the ascending aortic aneurysm and pericardium revealed important insights into the disease process. The presence of a pseudoaneurysm within the adventitia containing neutrophil polymorphs and the absence of stains for organisms in the aortic wall raised questions about the pathogenesis of the pseudoaneurysm, for which atherosclerosis may have played a significant aetiological role. Clinicians should remain vigilant about the possibility of atypical organisms causing these vascular conditions to improve patient outcomes.

This case underscores the importance of considering rare infectious agents like *S. constellatus* and Parvovirus B19 in the differential diagnosis of aortitis and aortic aneurysm, especially when blood cultures are negative. Advanced molecular diagnostics can aid in the timely identification and treatment of these unusual pathogens, potentially preventing life-threatening complications.

In summary, this is the third documented case of an infected aortic aneurysm caused by *S. constellatus* [9, 10]. This organism should be included in the list of pathogens causing mycotic aneurysms.
